# Morphological plasticity in a calcifying modular organism: evidence from an *in situ* transplant experiment in a natural CO_2_ vent system

**DOI:** 10.1098/rsos.140413

**Published:** 2015-02-11

**Authors:** Chiara Lombardi, Silvia Cocito, Maria Cristina Gambi, Paul D. Taylor

**Affiliations:** 1 ENEA Marine Environment Research Centre, PO Box 224, La Spezia 19100, Italy; 2 Laboratory of Functional and Evolutionary Ecology, Stazione Zoologica Anton Dohrn, Villa Comunale, Naples 80077, Italy; 3 Department of Earth Sciences, Natural History Museum, Cromwell Road, London SW7 5BD, UK

**Keywords:** modularity, morphological plasticity, vent system, bryozoan, ocean acidification, seawater temperature

## Abstract

Understanding is currently limited of the biological processes underlying the responses of modular organisms to climate change and the potential to adapt through morphological plasticity related to their modularity. Here, we investigate the effects of ocean acidification and seawater warming on the growth, life history and morphological plasticity in the modular bryozoan *Calpensia nobili*s using transplantation experiments in a shallow Mediterranean volcanic CO_2_ vents system that simulates pH values expected for the year 2100. Colonies exposed at vent sites grew at approximately half the rate of those from the control site. Between days 34 and 48 of the experiment, they reached a possible ‘threshold’, due to the combined effects of exposure time and pH. Temperature did not affect zooid length, but longer zooids with wider primary orifices occurred in low pH conditions close to the vents. Growth models describing colony development under different environmental scenarios suggest that stressed colonies of *C. nobilis* reallocate metabolic energy to the consolidation and strengthening of existing zooids. This is interpreted as a change in life-history strategy to support persistence under unfavourable environmental conditions. Changes in the skeletal morphology of zooids evident in *C. nobilis* during short-time (87 days) exposure experiments reveal morphological plasticity that may indicate a potential to adapt to the more acidic Mediterranean predicted for the future.

## 2. Introduction

Most animals are modular at one or more levels of organization [1]. Modularity can be reflected at the organismal, organ system and cellular levels, and in colonial animals also at the level of the individual units (zooids) making up the colonies. Modular colonies originate by the iterated replication of individual modules [2], which share resources and partition predation risk among themselves [3]. Growth, reproduction, response to threats or recovery from localized damage are facilitated by metabolic interchange, communication and cooperation between individual units, which play an essential role in colony functioning, even though modules often have the capacity to feed and reproduce independently [4,5]. The proximity and interconnections between different modular zooids enable a high level of integration of different parts of the colony, as observed in some bryozoan and cnidarian colonies [6,7].

When faced with new or altered environments (e.g. heterogeneous distribution of resources in time or space), organisms are able to change their phenotype to optimize their growth and reproductive output. This ability of a given phenotype to respond to changing conditions (i.e. phenotypic plasticity) [8] should promote colonization of and persistence in new or altered environments. In the long-term, phenotypic plasticity can be the target of natural selection under certain conditions, resulting in evolution through genetic assimilation or phenotypic accommodation [9]. Environmentally induced, non-heritable variation, such as phenotypic plasticity or learning, can be initially established in a population and later become genetically ‘assimilated’ such that the environmental stimulus previously required to produce the trait is no longer required [10,11]. Different types of phenotypic plasticity can contribute uniquely to adaptive evolution. Plasticity in morphology should be of particular benefit to sessile organisms that must tolerate environmental change without the ability to move away [12,13]. One such example in colonial organisms is adjustment in growth-form based on resource availability, which can enhance or maximize the acquisition of resources in heterogeneous environments [14].

Among modular organisms, bryozoans are a good group for investigating evolutionary ecology because of their extensive fossil record [15], often well-defined morphospecies exhibiting evolution in a pattern corresponding to punctuated equilibrium theory [16,17] (i.e. the sudden appearance of new species in the fossil record followed by long periods of species stability or stasis), preservation of past competitive interactions [18], and their high diversity and dominance in many shallow marine environments at the present-day. With respect to morphological plasticity in bryozoans, experiments carried out under controlled and natural conditions on various species have shown that different ambient temperatures can induce size changes in the zooids [19,20]. Laboratory simulations of temperature and pH levels expected to occur for the year 2100 and 2300 have shown that in one species—*Celleporella hyalina*—pH has a progressive and quasi-linear effect on species growth and reproductive investment [21]. A detrimental effect of reduced pH on individual growth was hypothesized, corroborating earlier evidence that pH can alter reproductive processes in other marine invertebrates [22,23]. In another bryozoan—*Schizoporella errata*—growing within natural CO_2_ vent systems, reallocation of energy from the production of defensive zooids (avicularia) to feeding zooids (autozooids) apparently occurred in low pH conditions close to the vents (i.e. low pH) [24].

Morphological responses, their functional significance and fitness implications are key to understanding the evolution of developmental plasticity in modular organisms (i.e. changes in size and shape of body structures at all hierarchical levels of organization) [25]. As developmental plasticity can be considered ‘as an integrated suite of adaptive solutions to environmental challenges in which the size and number of structures can respond to environmental cues’ [25], p. 203, studies on how different components of growth are modulated by environmental variables during development [21,26–28] are essential for making predictions on the survival and evolution of organisms in a changing world.

Since the industrial revolution, a rise of approximately 30–40% in atmospheric carbon dioxide (CO_2_) levels has resulted from increased anthropogenic activities such as fossil fuel combustion and deforestation [29,30]. Absorption of anthropogenic CO_2_ by the oceans—ocean acidification (OA)—during the past 150 years has caused the pH of the ocean to be reduced by 0.1 units simultaneously with a rise in global surface temperatures of 0.76°C [31]. Predictions based on fossil fuel utilizations suggest an additional pH decline of 0.3–0.5 units (750 ppm), and a subsequent pH drop of 0.7 units (3000 ppm) by 2300 [29,31]. The challenge currently facing scientists is to predict the long-term implications of OA and seawater warming (SW) for the diversity of marine organisms and for the ecosystem functions this diversity sustains. There is huge uncertainty in the extent to which adaptation or acclimation of organisms will mitigate the long-term effects of climate change and it is still a matter of debate if and how marine functional diversity will be impacted within the range of climatic changes, particularly OA, predicted for the coming centuries [32].

The aim of the current paper is to investigate the effects of OA and SW on the skeletal morphology of the Mediterranean bryozoan *Calpensia nobili*s (Esper, 1796). This species is analysed at zooid- and colony-level in order to: (i) investigate growth and morphology of zooids and colonies under different pH and temperature conditions; (ii) formulate developmental models for normal and altered environmental conditions; and (iii) assess species-level resilience to environmental changes (i.e. OA and SW). A transplantation experiment was conducted in a shallow Mediterranean volcanic CO_2_ vent system that naturally simulates pH values expected for 2100 [33]. Morphological and structural development of zooids, colony sizes and growth rates of colonies exposed to ‘normal’ (control) and ‘low’ pH (vent) conditions for 16, 34, 48, 57 and 87 days were investigated using an integrated system of image analysis and scanning electron microscopy (SEM). Growth models describing colony development under different environmental scenarios are developed from this data. The study thus contributes to the currently limited understanding of biological processes underlying the responses of modular organisms to global climate change and their potential for adaptation through morphological plasticity related to their modular construction.

## 3. Material and methods

### 3.1 The bryozoan *Calpensia nobilis* (Esper, 1796)

*Calpensia nobilis* is an encrusting bryozoan widespread in the Mediterranean. It is also recorded in the eastern Atlantic southwards along the northwest African coast and northwards to the Gulf of Saint-Malo and the Channel Isles [34,35]. Occurring from 10 to 30 m in depth, single-layered or multilayered encrusting colonies grow on several types of hard substrates, including rocks, shells, man-made objects, such as glass and ceramics, as well as other marine animals and plants [27,34,35].

The first zooid of the colony, the ancestrula, originates through settlement and metamorphosis of a sexually produced larva. Budding of new modular clonal zooids begins from the ancestrula. The rectangular or subhexagonal zooids are arranged in linear series. Unlike many related cheilostome bryozoans, *Calpensia* lacks defensive polymorphs (avicularia) and external brood chambers (ovicells). Each zooid has a D-shaped orifice and an extensive, porous frontal shield that is composed of an inner layer of calcite and an outer layer of aragonite [36] secreted beneath the cover of an epistegal coelom. Zooid length (ZL) ranges from 590 to 720 μm, orifice length (OL) 80 to 100 μm and orifice width (OW) 120 to 140 μm [34]. Young colonies are usually white, thin unilaminellar patches that can subsequently develop multilayered, three-dimensional architectures of cylindrical or nodular shape. Multilayered growth originates via self-overgrowth from a single colony or by overgrowth of several colonies [35,37]. Colony growth can be influenced by external factors, such as the presence of obstacles on the substrates or breakage inducing reparative growth.

Despite being recognized as one of the most important Mediterranean bioconstructors forming three-dimensional colonies several centimetres long on *Posidonia oceanica* (L.) Delile rhizomes and other substrates, there is no available data on growth rates in *C. nobilis*. Some *Calpensia* bioconstructions, which are favoured by strong hydrodynamic conditions, have been estimated to live for at least a decade [27]. The growth strategy of *C. nobilis* has led it to become a dominant species in the *P. oceanica* habitat, influencing the growth of seagrass rhizomes and, in the long-term, determining their death. The bimineralic composition of its skeleton, composed of approximately 60% calcite and 40% aragonite [38], makes it potentially susceptible to the effects of OA given the differential solubility of these two polymorphs of calcium carbonate.

### 3.2 Sampling and animal preparation

**Figure 1.**
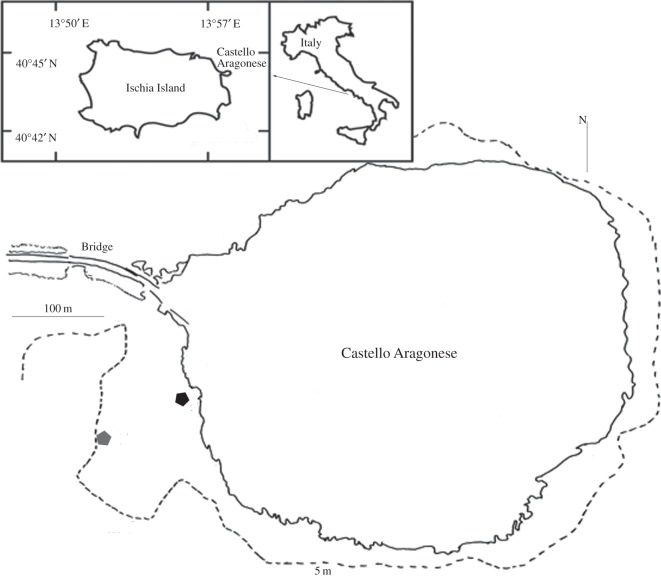
Study area and transplant sites around the CO_2_ vents off Ischia (Naples, Italy). Isobaths are represented as dotted lines. Grey and black symbols indicate the positions of control and vent (low pH) sites, respectively.

Fieldwork was carried out from the 7 June to 8 September 2009 off the southern coast of Ischia Island, Italy (40°41.31^′^ N, 13°53.36^′^ E) (figure 1). Colonies of *C. nobilis* were originally collected by means of scuba diving from a meadow located at 15 m depth around a rocky bank (Secca delle Formiche di Vivara) located in the Ischia channel between the islands of Vivara and Ischia and characterized by a strong current regime [27]. Thirty *P. oceanica* rhizomes bearing *C. nobilis* colonies were transported to the laboratory and placed in 20 l aquaria with a seawater turnover rate of 50% h^−1^. Colonies were removed from the rhizomes, washed and brushed gently to remove epibionts and sediment. Forty flat colonies with diameters of 3 cm and active growing edges were selected for the experiment. Each colony was attached to a tagged plastic plate using epoxy glue (HoldFast, USA) [24,39,40], mounted on a PVC plate and distributed between four cages (30×50 cm) with 10 colonies per cage.

### 3.3 Volcanic CO_2_ vent system and experimental set-up

Four cages containing *C. nobilis* colonies were transplanted into an area of natural volcanic CO_2_ vents where a natural gradient of seawater pH pertains. The vent area is located on the south side of Castello Aragonese (40°43.84^′^ N, 13°57.08^′^ E), off the southern coast of Ischia Island. It is characterized by a *P. oceanica* meadow located on a shallow rocky bottom [33,41]. Interestingly, *C. nobilis* has been found recently forming bioconstructions on *Posidonia* rhizomes on the north side of the Castello, close to the vent system (M. C. Gambi 2013, personal observation). Within the meadow, two suitable sites were selected for positioning the cages at 3–4 m depth at the endpoints of a 200 m long gradient of pH/CO_2_ (normal pH, vent sites/low pH), based on descriptions from previous studies [24,33,39,42]. Owing to the limited spatial extension of the vent area (especially in the low pH zone), and because other nearby vent sites differ substantially in venting rate and local current velocity [39], it was not possible to deploy more than two cages (2 m apart) per site. Each cage was fixed to a 30 kg concrete block to prevent movement and colony breakage. Two cages were deployed in normal pH (min: 7.90, max: 8.17, mean: 8.10), and two closer to the vent site (pH min: 6.89, max: 8.16, mean: 7.83) [24]. Colonies deployed on 7 June 2009 were collected from each site after 16, 34, 48, 57 and 87 days of exposure.

### 3.4 Environmental data

Seawater temperature data were obtained using two Hobo Onset loggers, fixed to the concrete blocks close to the cages, collecting data at 15 min intervals for the entire duration of the experiment. For pH measurements, water samples were collected between 10.30 and 12.00 in 250 ml polyethylene jars at 1 m depth, 16 times over the entire period of study. Three replicates of seawater samples were collected per treatment at each date and seawater temperature was measured *in situ*. Jars were transported to the laboratory in a coolbox in order to maintain temperature conditions. Here, pH_*T*_ and temperature were measured using a pH-meter (pH 300, Hanna Instrument, USA) (±0.01 pH unit) and associated thermometer within 2 h of collection. The pH-meter was calibrated using standard reference buffers for pH 4.00, 7.00 and 10.00 at 25°C (Sigma-Aldrich, Canada). All laboratory pH data were then adjusted to seawater temperature values, measured *in situ*, using Gieskes' formula (1969) (pH=pH_1_+0.0114[*t*_1_−*t*_*m*_], where pH1 is the pH measured in the laboratory by the instrument, *t*_1_ is the measuring temperature and *t*_*m*_ is the *in situ* temperature).

At the control site, mean pH was 8.09 (min 8.07±0.09 at day16; max 8.10±0.06 at day 87), whereas at the vent site, mean pH was 7.76 (min 7.72±0.46 at day 48; max 7.83±0.41 at day 87) [24]. During the study period, mean seawater temperature was 25.6±0.8°C at the control site (24.7±0.7 at day 16; 27.1±0.8 at day 57) and 25.3±0.8°C at the vent site (24.3±0.6 at 16 day; 25.7±0.9 at day 87). Seawater temperatures were not significantly different between the two sites but increased slightly (by *ca* 2°C) during the study period at both sites (*t*-test) [24].

### 3.5 Growth, life history and morphological plasticity

Prior to transplantation, the colonies of *C. nobilis* were placed on sheets of graph paper and photographed using a digital camera (D70, Nikon, Japan) fixed to a tripod in order to maintain the same distance between the camera and the samples. Further images (two colonies randomly taken per duplicate cage from each of the two sites, i.e. four colonies per site) were taken after 16, 34, 48, 57, 87 days. In order to quantify colony growth, colony areas (cm^2^) were measured using CPCe software. Only colonies with newly formed edges grown on flat surfaces were considered.

After final collection, live colonies of *C. nobilis* were soaked for several days in a solution of 20% sodium hypochlorite to remove organic material, washed thoroughly in water and dried. Scanning electron microscopes (LEO 1455VP and EVO LS 15, Zeiss, Germany) were used to examine the uncoated specimens at magnifications ranging from 65 to 1000×. Both newly formed zooids at the growing edge and zooids from older parts of the colonies were imaged for samples after treatment periods of 16, 34, 48, 57 and 87 days. Environmentally induced changes in skeletal growth patterns indicative of morphological plasticity were studied using SEM imagery of colony growing edges at a standard magnification of 65×.

### 3.6 Data analyses

To examine differences in colony growth through the study period, colonies were photographed at the beginning of the experiment and two colonies per duplicate cage from each of the two sites were photographed again at the end of each time interval (16, 34, 48, 57, 87 days). Colony growths (i.e. colony areas) were compared by using two-way ANOVA (Statistica).

Interaction (treatment × exposure time) was considered. Because new colonies were sampled at each time interval all the analysed samples were independent. Δ (Delta) colony growth (i.e. mean colony growth at period B - mean colony growth at period A) was computed for each time interval. When ANOVA indicated significant differences, the source of the difference of one of the two factors (treatment or exposure time) was identified using a Student–Newman–Keuls (SNK) test. Prior to analysis, a Cochran's test was employed to assess the homogeneity of variance.

In order to study life-history strategies, lengths and widths of 20 zooids and primary orifices from each of the two colonies per duplicate cage sampled at each time period from each of the two sites were measured from digital scanning electron micrographs using ImageJ. Only newly formed but complete adult zooids were considered for this analysis in order to avoid any variability among zooids due to different stages of development [6,19,20,43–49]. Also discounted from the analysis were zooids at row bifurcations and zooids with distorted shapes due to growth on irregular surfaces, at junctions between different colonies, or formed from repair after colony breakage [35,45,46]. Two-way ANOVA was used to test difference in zooid morphometrics between treatments and among periods. When ANOVA indicated significant differences, the sources were identified using a SNK test. Prior to analysis, a Levene's test was employed to assess the homogeneity of variance. When data were heterogeneous, data transformations (square root, logarithmic and arcsine) were applied to the raw measurements. The influence of pH and temperature on zooid morphometrics was analysed using ANCOVA. Prior to analysis, a Cochran's test was employed to assess the homogeneity of variance.

## 4. Results

### 4.1 Colony growth

**Figure 2.**
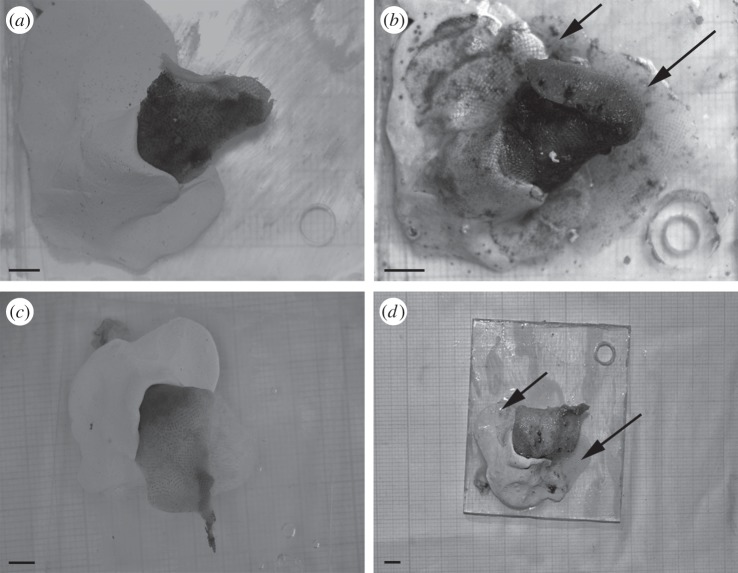
*Calpensia nobilis* colony growth. Colonies at control (*a*) and vent site (*c*) before the deployment; colonies at the control (*b*) and vent (*d*) sites after 87 days of exposure. Newly formed parts of the colonies are indicated by arrows. Scale bars, (*a*–*d*) 0.5 cm.

**Figure 3.**
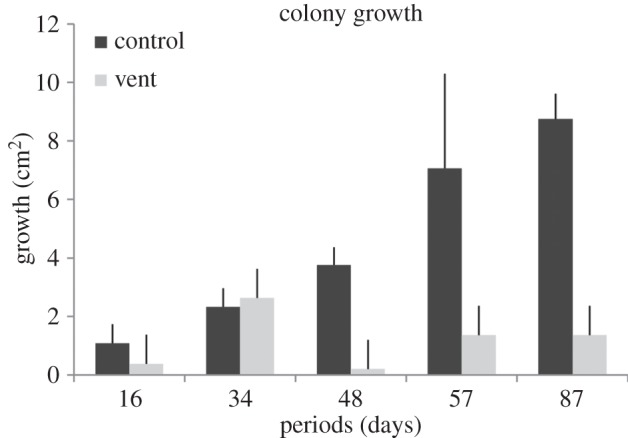
Mean growth in area ± s.d. (cm^2^) of *C. nobilis* colonies from the control (black bars, normal pH) and vent site (grey bars, low pH) after 16, 34, 48, 57 and 87 days of exposure.

Colonies of *C. nobilis* from the control site grew rapidly in size throughout the study, spreading onto the mounting epoxy while extending upright branches (figure 2*a*,*b*). Colony size increased from 1.09±0.65 cm^2^ after day 16 to 8.75±0.87 cm^2^ after day 87 (figure 3). Interestingly, Δ colony growth was fairly constant throughout the study period (min 1.09 cm^2^ from day 0 to day 16; max 1.68 from day 57 to day 87), with the exception of the time interval between days 48 and 57 when colonies showed a Δ growth of 3.29 cm^2^ (figure 3). Colonies transplanted to the low pH vent site showed a different pattern. They grew regularly for the first two periods (to days 16 and 34), increasing in colony area by 0.36±0.04 cm^2^ and 2.85±0.67 cm^2^, respectively. After 48 days of exposure, colonies experienced their lowest increase in area (0.26±0.18 cm^2^). The lowest Δ colony growth was also between days 34 and 48 (−0.04 cm^2^), followed by between days 57 and 87 (−0.1 cm^2^), while the highest was recorded between days 16 and 34 (2.49 cm^2^) (figure 3). During other periods, colonies from the low pH site grew approximately half the rate of those from the control site.

Colonies maintained under control conditions grew more on average than colonies exposed to low pH vent conditions (table 1); differences in growth were found between time intervals in both conditions, with a significant decrease in colony growth after 57 days of exposure in vent conditions (SNK test: 16=34=48>57=87) and an increase in control conditions (SNK test: 16=34=48>57=87) and the treatment × period interaction (table 1).

**Table 1.** Comparison among treatments for different time intervals of *C. nobilis* morphometrics using two-way ANOVA. (Total number of colonies (*N*)=40; total cages (*k*)=4 cages; colonies per cage (*nK*)=10. Measured zooid per colony (*Z*): 20; Tr: treatment (pH)(control: 8.10, 7.90–8.17; vent: 7.83, 6.89–8.15); Et: exposure time intervals (of unequal duration): Et_1_=16 days (Et_0_+16), Et_2_=34 days (Et_1_+18), Et_3_=48 days (Et_2_+14), Et_4_=57 days (Et_3_+9), Et_5_=87 days (Et_4_+30). Significant values (*α*=0.05 or 0.01, when specified) in italic.)

		colony growth				zooid length				zooid width		
	d.f.	MS	*F*	*p*	d.f.	MS	*F*	*p*	d.f.	MS	*F*	*p*
Tr	1	85.8183	48.0228	*0*.*0000*	1	166 802	99.92	*0*.*0000*	1	2462	30.6300	*0*.*0000*
Et	4	17.8260	9.9752	*0.0001*	4	40 850	24.47	*0.0000*	4	2251	28.0000	*0.0000*
Tr × Et	1	16.5867	9.2817	*0.0002*	1	7554	4.53	*0.0016*	1	423	5.2500	*0.0005*
error	20	1.7870			191	1669			191	80		
Cochran *C*				*0.002*				*0.0000*				*0.0002*
transformation			none				none	*α*<0.01			none	*α*<0.01

### 4.2 Skeletal ontogeny

**Figure 4.**
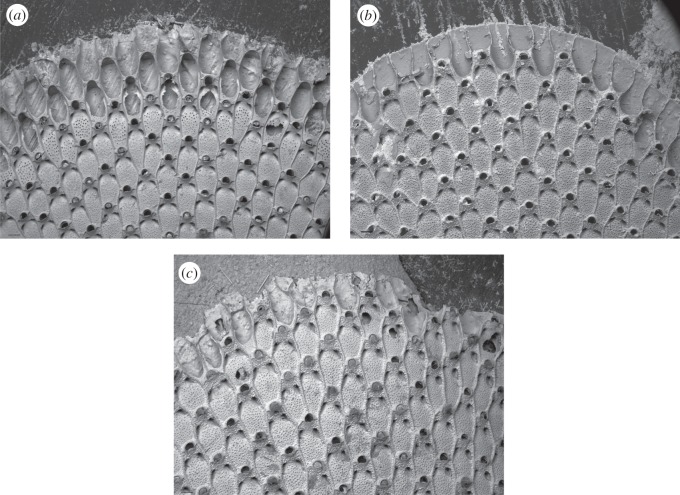
Scanning electron micrographs of *C. nobilis* colony growing edges from control and vent sites. (*a*) Colony exposed to control conditions for 34 days; (*b*) colony exposed to vent conditions for 57 days; (*c*) colony exposed to vent conditions for 87 days. Scale bars, 200 μm.

**Figure 5.**
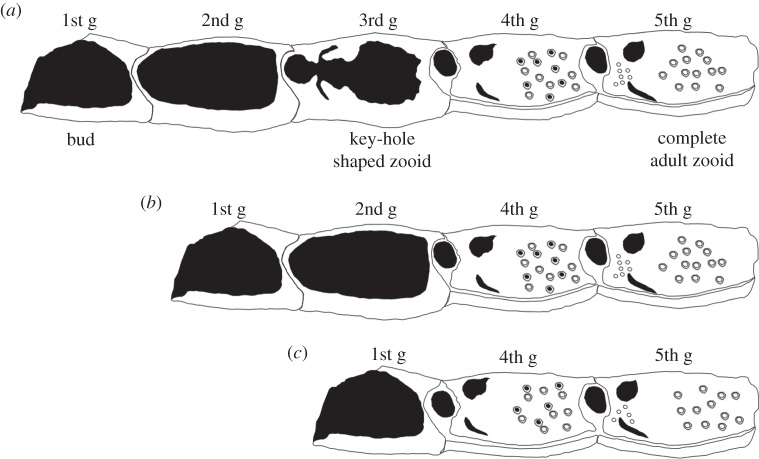
Diagram of morphological plasticity at the growing edge. (*a*) Normal zooidal development at the growing edge: from the first (new buds) to the fifth generation (complete adult zooids). Altered zooidal development at the growing edge in colonies exposed at vent conditions for 57 days (*b*) and 87 days (*c*). Note the lack of the second and third zooid generations.

Scanning electron micrography of growing edges (figure 4) revealed regular colony growth and well-developed zooids in specimens exposed to vent conditions for 16, 34 and 48 days. In the control, the first generation zooids (i.e. latest buds to be formed) were usually characterized by the beginnings of the basal and lateral walls; second generation zooids had more complete lateral and basal walls; third generation zooids showed incomplete zooidal skeletons with frontal walls partially formed and ‘keyhole-shaped’ orifices; fourth generation zooids were almost complete, but with the gymnocyst sometimes missing and the frontal wall not entirely calcified; and fifth generation zooids typically had complete skeletons (figure 5*a*). Giant and irregular zooids were observed within the recently formed parts of some colonies growing over irregular surfaces produced by the presence of resin on the plastic slides.

Normal growth characterized colonies from vent sites after 16, 34 and 48 days of exposure, but after 57 days colonies displayed a reduced number of zooid generations, with the third generation zooid stage as defined above being absent (figure 5*b*). After 87 days of exposure at the vent site, second generation zooids were also missing (figure 5*c*).

The older parts of colonies (i.e. those formed prior to gluing onto the resin) showed skeletally mature zooids with thickened frontal walls and well-defined zooidal boundaries. Traces of corrosion of the gymnocysts and primary orifices were observed after 16 days of exposure in colonies deployed at the low pH vent sites; after 34 and 48 days, the primary orifices were thoroughly corroded, as were the gymnocysts around them, although those parts of the gymocyst connected to the lateral walls were preserved. After 57 days, the shapes of the primary orifices were strongly modified, being more rounded than is usual, and gymnocysts were lacking. After 87 days, pseudopores piercing frontal walls were enlarged and the frontal walls themselves corroded along the zooidal boundaries, progressively more so with longer exposure times.

### 4.3 Morphometric variability

**Figure 6.**
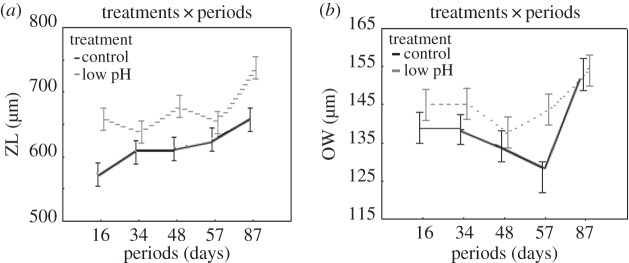
Mean zooid length (ZL) (μm) (*a*) and orifice width (OW) (μm) (*b*) within each treatment (black-entire line: control; grey-dashed line: vent) across periods (16 days, 34 days, 48 days, 57 days, 87 days). Vertical bars denote 0.95% confidence intervals.

SEM imagery of the growing edges of all colonies allowed 20 mature zooids to be measured for ZL, OL and OW. Mean ZL was 613.21±28.5 μm at the control site and 673.46±38.7 μm at the low pH vent site. Two-way ANOVA revealed significant differences in ZL between treatments (table 1), among periods in ZL and period × treatment (SNK for the control: 16 day results were different from 34, 48, 57 and 87 days; vent conditions: 87 day results differed from all the other periods) (table 2 and figure 6*a*). The mean value of OW was 138.8±9.9 μm at the control site and 143.8±8.1 μm at the vent site. Two-way ANOVA revealed differences between treatments, among periods and treatments × periods (table 1) (SNK for the control: 57 and 87 day results differed from all the other periods; for vent conditions: 87 day results differed from all other periods) (figure 6*b*). ANCOVA showed ZL to be significantly related to pH (*F*_1_=5.98,*p*=0.02), while temperature had no significant effect (*F*_1_=1.1,*p*=0.28); on the other hand, OW was not significantly related to pH (*F*_1_=0.8,*p*=0.38) but was significantly related to temperature (*F*_1_=6.7,*p*=0.01).

**Table 2.** Mean zooid length and primary orifice width ± s.d. (μm) of *C. nobilis* colonies from the control and vent site after 16, 34, 48, 57 and 87 days of exposure (exposure time intervals). (Measurements were made on 20 zooids per colony (8) per exposure time intervals (5).)

exposure time (d)	treatment	mean zooid length ± s.d.	mean orifice width ± s.d.
16	control site	587.29±28.11	144.68±9.25
	vent site	657.83±31.34	144.69±9.25
34	control site	601.82±30.89	137.04±10.44
	vent site	638.18±38.46	145.30±10.07
48	control site	593.36±50.29	134.57±14.64
	vent site	680.17±56.90	131.32±31.17
57	control site	627.33±15.08	125.76±6.63
	vent site	653.83±34.89	143.63±9.96
87	control site	656.26±32.05	151.93±7.19
	vent site	737.27±23.69	153.92±9.79

## 5. Discussion

The ability of an organism to respond phenotypically to changing conditions (i.e. phenotypic plasticity [8]) should enhance its ability to colonize and persist in new environments. In the long-term, phenotypic plasticity is a developmental process that can be the target of natural selection and—under certain conditions—will produce evolutionary changes through genetic assimilation or phenotypic accommodation [9]. The current study has shown that the exposure of colonies of the bryozoan *C. nobilis* to reduced pH results in phenotypic changes, suggesting potential plasticity of the species to pH conditions predicted for the future. In current conditions (i.e. control), colonies showed progressive and constant growth, with a peak growth rate (Δ growth: 3.29 cm^2^) occurring between 48 and 57 days (June–August). This peak is hypothesized to reflect either progressive ‘acclimatization’ of the colonies to the experimental set-up or to the optimal food abundance between days 48 and 57. Future studies on food availability are needed to assess these alternatives, although changes in food availability have been reported to determine changes in colony growth and survival in natural conditions in another species of bryozoan [43]. The drop in Δ growth observed after 57 days could be due to the higher seawater temperature (mean value: 25.7±0.8°C), which can negatively affect bryozoan growth and calcification [39,40]. Colonies from the low pH vent site showed the lowest Δ colony growth (−0.04 cm^2^) between 34 and 48 days, and a significant decrease in growth between days 57 and 87. Between days 34 and 48, colonies reached a possible ‘threshold’ due to the combined effects of exposure time and pH.

As modular organisms, bryozoans can be analysed at two different hierarchical levels: the colony as a whole and its constituent zooids [2,4,5,50]. Processes leading to colony growth involve zooid formation, development and completion [45,46], and these may vary among species. In *C. nobilis*, colonies from normal conditions show four generations at the growing edge, ranging from first generation zooids (new buds) with developing basal and lateral walls, to the skeletally mature fifth generation zooids. The presence of giant or irregular zooids in a few colonies could be due to the resin on the plastic slides as growth in *C. nobilis* is known to be highly susceptibility to any obstacles on the substrate [35]. This characteristic, as well as the potential of the species to undergo repair and regeneration after damage and to develop rapid budding in localized areas (i.e. ‘giant bud’ formation and transition from ‘normal’ to ‘multizooidal’ budding processes), helps to explain the dominance of *C. nobilis* over other encrusting species in natural environments.

In low pH vent conditions, colonies seem to prioritize the completion of already-formed zooids over the formation of new zooids. Between 48 and 57 days of exposure, these colonies show a reduced number of zooid generations, with the third generation zooids missing (figure 5*b*), a trend that also continues after 87 days of exposure when two generations of zooids (third and second) are absent (figure 5*c*). It is likely that these colonies are responding to the multiple stressors of high temperature, low pH and suboptimal food availability by investing less energy in budding new zooids. Instead, they seem to reallocate metabolic energy towards the consolidation and strengthening of existing zooids. This modification can be hypothesized as a change in life-history strategy to support persistence under unfavourable environmental conditions.

Bryozoan colonies conserve changes in the environment experienced during their lifetimes. For example, zooid size is inversely proportional to the ambient temperature at the time of budding and hence intracolony variation in zooid size scales with seasonal variability in seawater temperature [19,20]. Zooid length is more sensitive to temperature than zooid area and width [47,48]. Our colonies of *C. nobilis* do not reveal a relationship between zooid length and temperature. However, in contrast to previous studies that have investigated species over long timescales [49,51,52], our data on zooid length were collected over the summer months only. Falling within the range of values reported in literature for the species (*ZL*=590–720 μm, [34]), other factors such as food quantity or size possibly acted as drivers in determining zooid length during summer. Interestingly, colonies exposed to low pH vent conditions produced longer zooids and wider primary orifices, as has been observed previously in another bryozoan—*Schizoporella errata*—which was exposed to the same conditions [24]. Although longer zooids with wider primary orifices were produced in low pH vent conditions, in terms of energy balance within the colony, reduced growth and less investment in zooid production is suggested with the energy budget of the colony being reallocated to the existing zooids, making them more resilient under unfavourable ambient conditions of low pH and potentially enabling enhanced species persistence at the vent site. In the oldest part of the colonies, where the organic cuticle is missing or degraded and the mineralized skeleton more exposed, low pH has an impact on the most vulnerable aragonitic parts of the skeleton which increases with exposure time. Zooids show traces of corrosion on the gymnocysts and primary orifices after 16 days of exposure. After 87 days of exposure, orifices are altered in shape, gymonocysts are missing, pseudopores enlarged and the frontal wall corroded along the zooidal boundaries.

Morphological plasticity and its functional significance and fitness implications are key to understanding the evolution of developmental plasticity in modular organisms [25]. After a short-time exposure under unfavourable, low pH conditions, *C. nobilis* shows changes at the zooidal level suggesting morphological plasticity that may indicate a potential to adapt to the more acidic Mediterranean predicted for the future. Indeed, recent observations by one of us (M. C. Gambi 2013) show the presence of *C. nobilis* in the *Posidonia* meadow in normal pH conditions but less than 30 m from the vent area on the north side of the Castello Aragonese. This represents a sign of range expansion in this species. Because developmental plasticity can be considered ‘as an integrated suite of adaptive solutions to environmental challenges in which the size and number of structures can respond to environmental cues’ [11,25, p. 203], [50], future studies, including longer time transplants and cross-transplants should be performed in vent systems and under controlled conditions, coupled with molecular investigations. Such studies would provide evidence on how different components of growth are modulated by environmental variables during development, and whether environmentally induced, non-heritable variation can become genetically assimilated in bryozoans. With this information, better predictions could be made of organism survival and evolution, and thus ecosystem changes, loss or resilience in a changing world.

## Data accessibility

The original data has been deposited in Dryad Digital Repository http://dx.doi.org/10.5061/dryad.303j3 an and they will be available after the publication of the manuscript.
